# Sexual health in female and male cancer survivors – compared with age-matched cancer-free controls in Norway

**DOI:** 10.2340/1651-226X.2025.42451

**Published:** 2025-03-07

**Authors:** Emilie Åsberg, Guro F. Giskeødegård, Jarle Karlsen, Cecilie E. Kiserud, Guro Aune, Marianne Nilsen, Randi J. Reidunsdatter

**Affiliations:** a Department of Circulation and Medical Imaging, Faculty of Medicine and Health Sciences, Norwegian University of Science and Technology (NTNU), Trondheim, Norway; b Department of Public Health and Nursing, Faculty of Medicine and Health Sciences, NTNU, Trondheim, Norway; c Department of Oncology, St. Olav’s Hospital, Trondheim University Hospital, Trondheim, Norway; d Department of Clinical and Molecular Medicine, Norwegian University of Science and Technology (NTNU), Trondheim, Norway; e Department of Oncology, Oslo University Hospital, Oslo, Norway; f Section of Gynecologic Oncology, Department of Obstetrics and Gynecology, St Olav’s Hospital, Trondheim University Hospital, Trondheim, Norway; g Department of Social Work, Faculty of Social and Educational Sciences, NTNU, Trondheim, Norway

**Keywords:** Sexual health, sexual dysfunction, cancer survivors, EORTC QLQ-SHQ22, QLQ-BR23, QLQ-BR45, cancer-free controls, patient-reported outcomes, PROM

## Abstract

**Background and purpose:**

Sexual dysfunction is a common late effect of cancer reducing quality of life. This study investigated sexual health in cancer survivors shortly after diagnosis and at long-term follow-up compared to the general population.

**Methods:**

A nationwide survey stratified by sex and age was distributed to a representative sample of the Norwegian population. Of the 5,135 respondents (33% response rate), 453 were cancer survivors, and 4,682 were cancer-free controls. Time since cancer diagnosis was divided into two categories: 2 years or less (short-term) and over 2 years (long-term). Sexual health was evaluated using the EORTC questionnaires SHQ-22 and the sexual domains of the QLQ-BR23/QLQ-BR45. Multivariable linear regression analyses compared sexual health between cancer survivors and cancer-free controls, and between short- and long-term cancer survivors.

**Results:**

Cancer survivors reported significantly poorer sexual health outcomes than cancer-free controls, except for the importance of maintaining a sexually active life, rated equally important. There were minimal differences in sexual health between short-term and long-term cancer survivors. Interestingly, male cancer survivors appeared to be more affected by sexual health challenges than females, when compared to their cancer-free controls.

**Interpretation:**

This study is the first to utilize the EORTC SHQ-22 questionnaire to assess sexual health in cancer survivors and controls. Sexual health was found to be significantly worse in cancer survivors compared to age-matched controls. It is imperative to address this overlooked health issue in the follow-up programs for cancer survivors.

## Background

Sexual dysfunction is a common late effect of cancer treatment [1–4], encompassing conditions such as hypoactive sexual desire and arousal issues, orgasmic dysfunction, ejaculatory dysfunction, and sexual pain-penetration disorder. To receive this diagnosis, these challenges must persist over an extended period (several months), occur frequently and be accompanied by clinically significant distress (IDC-11) [5]. Sexual dysfunction can be a result of both biological, physiological, physical but also psychological changes associated with cancer itself and its treatment, and it may present differently in women and men. The risk of developing sexual dysfunction is associated with hormonal changes, aging, depression comorbidities, chronic medication use, malignancy, and exposure to toxics [6–8].

Prostate cancer is the most prevalent cancer in men [9], while testicular cancer is the most common cancer diagnosis in younger men (15–35 years) [10]. Both cancers have high survival rates [11, 12] and are strongly linked to sexual dysfunction [3, 4]. Decreased libido, erectile issues, and problems with ejaculation and orgasm are common in male cancer survivors, exacerbated by side effects of cancer treatment, like hypogonadism, fatigue, fear of incontinence, and psychological distress [3, 4, 13]. Erectile dysfunction affects 43% of male cancer survivors following the completion of treatment, according to a large systematic review [14]. Breast cancer, the leading cancer diagnosis in women, and gynecological cancers are also associated with sexual dysfunction [2], with women with breast and cervical cancer having a 3.5- and 2.7-fold increased risk of sexual dysfunction compared to those without cancer [1]. Female sexual dysfunction, which can involve recurring difficulties with desire, arousal, lubrication, acheiving orgasm, or dyspareunia, is reported in as many as 70-80% of female cancer survivors [1].

The European Organization for Research and Treatment of Cancer has developed the first cancer-specific Sexual Health Questionnaire QLQ-SHQ22. The QLQ-SHQ22 can be used for both male and female cancer survivors regardless of cancer type, in clinical and research settings [15]. It is based on the World Health Organization’s holistic definition of sexual health and has so far only been used in small studies [16, 17].

Even though cancer survivors are prone to sexual dysfunction, we know that sexual problems are also common in the general population, with a higher prevalence in women than men, and associated with increasing age and morbidity [18]. The gap of knowledge in this area is thus to assess sexual health in cancer survivors in comparison to age-matched individuals without cancer. This study aims to explore the sexual health of cancer survivors, both short- and long-term, among female and male survivors compared to cancer-free controls from the general population.

## Method

### Study procedure and participants

The data was obtained from a nationwide electronic and postal cross-sectional survey conducted in the Norwegian general population with the primary aim of establishing normative values for EORTC questionnaires QLQ-C30, -BR23/45 and -SHQ22 i.e the Normative study [18]. With permission from the Norwegian Tax Administration, the sample was stratified by sex and age groups (18–29, 30–39, 40–49, 50–59, 60–69, 70–79 years) and randomly selected from the Norwegian National Register by the national IT company Evry (Evry.com). Information on morbidities, marital status, living arrangements, education, profession, employment status, and income was included in the survey.

As part of the preparatory phase, a pilot study involving 15 participants was conducted to evaluate the comprehensibility of the survey items and the digital platform eFORSK (https://www.klinforsk.no/info/Informasjon). Modifications were made based on the feedback received. To increase the response rate, the study was promoted to the general audience through social media, national radio news, podcasts, newspapers, blogs and external channels at the Norwegian University of Science and Technology (NTNU). The survey was released in autumn 2021, and participants were notified and informed through the national online health services “Helsenorge”, the digital mailbox “Digipost”, email or private messages. Individuals without digital access received the survey by postal mail. A reminder was sent to the digital responders after 2 weeks [18].

Of the 15,627 invited, 5,135 responded (33% response rate), and of these, 453 individuals reported being diagnosed with one or more types of the following cancer types: breast, prostate, intestinal, skin, gynecological, lymphoma, testicular, bladder, lung, leukemia, thyroid and others (*n* = 46). These individuals were classified as cancer survivors, while the remaining 4,682 individuals represented the cancer-free controls. The year of cancer diagnosis was self-reported, and time since diagnosis was divided into two categories: *2 years or less* (short-term) and *more than 2 years* (long-term) since the most recent diagnosis. The distinction between short- and long-term survivors was determined based on the time when patients were expected to resume normal life activities after completing the initial cancer treatment [19]. For sex-specific analyses in the cancer survivors, breast-, ovarian-, cervix-, and uterus cancer defined the female cancers (*n* = 127), while prostate- and testicular cancer defined male cancers (*n* = 99).

### Measures and variables

Sexual health was assessed using the new EORTC questionnaire QLQ-SHQ22 [15]. It consists of eight functional scales measuring *sexual satisfaction* (among the sexually active), *importance of sexual activity* (with or without a partner), *libido, impact of treatment, communication with professionals about sexual problems, insecurity with a partner* (among those with a partner), *femininity* (women only), *masculinity and confidence with erection* (men only), and four symptom scales assessing the impact of *sexual pain, worry about incontinence, fatigue and vaginal dryness* (women only). The instrument has proven psychometric properties and is found applicable in research and clinical practice for assessing sexual health in survivors and patients, across diagnosis and stages of disease [20].

As the EORTC breast cancer module QLQ-BR23 has been extensively used in earlier studies, we included the sexual health scales from this module to be able to compare with previous research [21, 22]. In addition, we included sexual items from the newer and extended module QLQ-BR45, as it captures endocrine symptoms more comprehensively and thus complements the SHQ-22 [23]. Response options for all items ranged from *Not at all* (1) to *Very much* (4), with ability for responding *Not relevant* on three items asking whether disease and treatment have an impact on various life conditions.

Scales were calculated according to the EORTC scoring manual as the average score and transformed into a 0−100 scale, where higher scores indicate better functioning for functional scales and higher symptom burden for symptom scales [24]. Interpretation of clinically significant differences is well established for the EORTC QLQ-C30 scales; a mean score difference of 5–10 is regarded as small, 10–20 as moderate and above 20 as a large clinical difference [24], which we regarded applicable for the QLQ-SHQ22 and the QLQ-BR-modules due to the same scoring system.

*Satisfaction with weight* was used as a proxy for body mass index (BMI) (as height and weight were not collected) and was evaluated by one question ‘*Are you satisfied with your weight at the moment?*’ with five response options *Yes; No, a bit too heavy; No, too heavy; No, a little too light* and *No, too light.* The variable was dichotomized by combining the two categories of ‘too *heavy*’ into ‘*Overweight’* and the other three categories into ‘*Normal*’ *or ‘Underweight*’.

Morbidity was assessed using the *Self-Administered Comorbidity Questionnaire (SCQ)* [25]. The SCQ evaluates the presence of up to 15 health conditions, and assesses whether the person is receiving treatment, and whether the condition limits any activities or functioning. Morbidity was defined as *having one or more morbidities that limited daily activities/functioning (Yes/No)*.

### Statistical analyses

Characteristics of the samples were calculated using descriptive statistics. Mean values with standard deviation (SD) of the EORTC QLQ-SHQ22, QLQ-BR23 and QLQ-BR45 sexual items were calculated in five age groups (18–39, 40–49, 50–59, 60–69, and 70–79 years).

Multivariable linear regression models with adjustments, guided by specific directed acyclic graphs (DAGs) to select relevant confounders for the specific research questions in scope, were performed to compare cancer survivors and cancer-free controls, and for comparing sexual health in short-term and long-term survivors. Subanalyses were performed in the defined female- and male cancer survivors with comparisons to their controls (see DAGs in Supplementary Figure 1). Missing values were handled by excluding observations in ‘complete case analyses’, in accordance with the default procedures in SPSS version 29.

### Ethics

The study was approved by the Regional Committee for Medical Research Ethics (REK 2020/58888). Study information was provided with the survey, and completion was considered as giving informed consent.

## Results

### Participants

The study included 453 cancer survivors and 4,682 cancer-free controls. The mean age of cancer survivors was 63 years (SD = 11.5), while the cancer-free controls had a mean age of 48 years (SD = 16.2). Both samples had an almost equal proportion of men and women. The characteristics of the study participants are detailed in Table 1.

**Table 1:** Sample characteristics for the study population.

		Cancer survivors N = 453 (%)	Cancer-free controls N = 4669 (%)
Sex	female	225 (49.7%)	2508 (53.7%)
	male	228 (50.3%)	2161 (46.3%)
Age, mean (SD)		63.8 (11.5)	47.9 (16.2)
Age categories	18–29 years	6 (1.3%)	774 (16.6%)
	30–39 years	18 (4.0%)	800 (17.1%)
	40–49 years	31 (6.8%)	843 (18.1%)
	50–59 years	67 (14.8%)	948 (20.3%)
	60–69 years	164 (36.2%)	814 (17.4%)
	70-79 years	167 (36.9%)	490 (17.4%)
Education	Compulsory or less	32 (7.2%)	260 (5.6%)
	Junior high school (1-2 years)	114 (25.5%)	890 (19.1%)
	Junior and senior high school (1-4 years)	66 (14.8%)	1005 (21.6%)
	University degree (<4 years)	120 (26.8%)	1163 (25.0%)
	Postgraduate degree (>4 years)	115 (25.4%)	1334 (28.7%)
Employment status	Employed full-time	123 (25.5%)	2699 (57.8%)
	Employed part-time	37 (7.7%)	560 (12.0%)
	Homemaker	2 (0.4%)	88 (1.9%)
	Student	5 (1.0%)	408 (8.7%)
	Unemployed	2 (0.4%)	84 (1.8%)
	Retired	228 (47.3%)	773 (16.5%)
	Full time sick leave	16 (3.3%)	106 (2.3%)
	Part time sick leave	6 (1.2%)	68 (1.5%)
	Disability pension	59 (12.2%)	310 (6.6%)
	Occupational rehabilitation	4 (0.8%)	42 (0.9%)
Relationship status	Single/not in a steady relationship	47 (10.4%)	817 (17.6%)
	Married or in a steady relationship	338 (74.6%)	3404 (72.9%)
	Separated/divorced	38 (8.4%)	302 (6.5%)
	Widowed	30 (6.6%)	126 (2.7%)
Health status	Morbidities 1*	168 (37.1%)	1322 (25.8%)
	Morbidities 2**	303 (66.9%)	2970 (58.0%)
	Heart disease	26 (5.7%)	65 (1.4%)
	High blood pressure	17 (3.8%)	61 (1.3%)
	Pulmonary disease	31 (6.8%)	166 (3.6%)
	Migraine	18 (4.0%)	336 (7.2%)
	Diabetes	6 (1.3%)	46 (1.0%)
	Kidney disease	4 (0.8%)	14 (0.3%)
	Gastric ulcer or intestinal disease	12 (2.6%)	95 (2.0%)
	Arthrosis	97 (21.4%)	435 (9.3%)
	Epilepsy	3 (0.7%)	7 (0.1%)
	Stroke or cerebral hemorrhage	7 (1.5%)	20 (0.4%)
	Depression	29 (6.4%)	396 (8.5%)
	Other psychological issues	25 (5.5%)	373 (8.0%)
	Rheumatic disease	27 (6.0%)	210 (4.5%)
Weight satisfaction			
	Not satisfied	73 (16.2%)	910 (17.8%)
Time after cancer diagnosis (n= 434)	0 – 2 years	97 (22.3%)	-
	2 years +	337 (77.6%)	-
Female specific cancer	Breast-, ovarian-, cervix-, and uterus cancer	127 (56.4%)	
Male specific cancer	Prostate- and testicular cancer	99 (43.4%)	

* Morbidities 1 are based on the criteria of having one or more of the below listed health conditions affecting daily activity.

** Morbidities 2 are based on the criteria of having one or more of the same below listed health conditions. Employment status and Morbidities (including cancer types) allows for multiple responses.

### Differences in sexual health between cancer survivors and cancer-free controls

**Figure 1:**
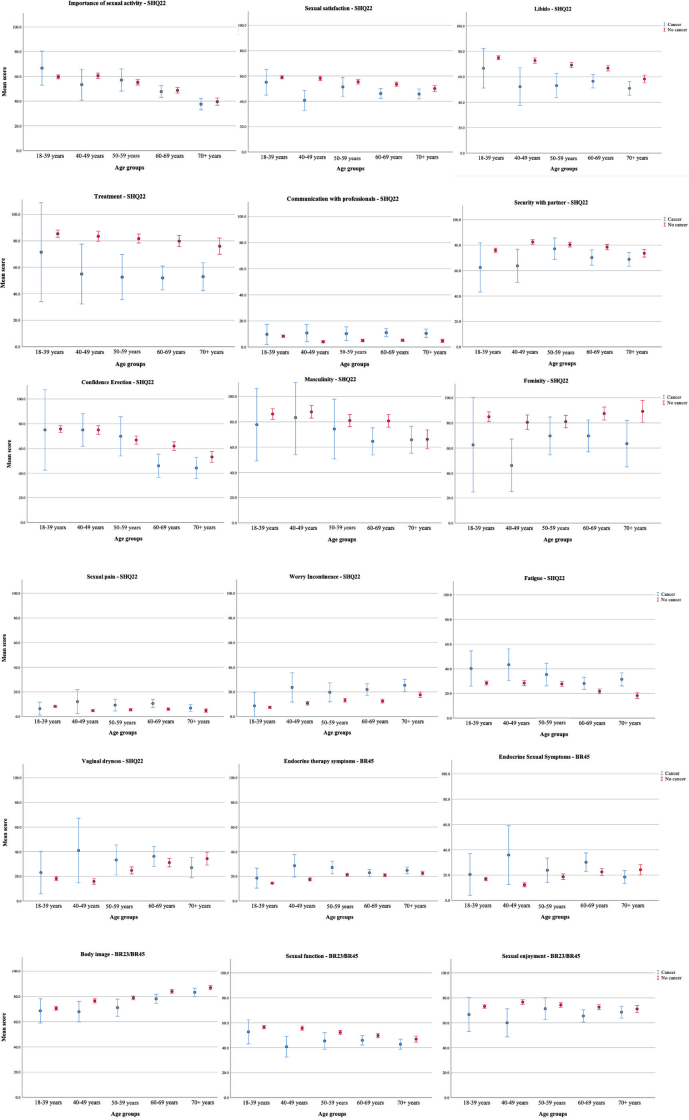
Displays the average scores on the sexual health questionnaire EORTC QLQ-SHQ22, along with sexual items in the modules QLQ-BR23 and QLQ-BR45 for cancer survivors and age-matched cancer-free controls.

Mean values of the EORTC QLQ-SHQ22, QLQ-BR23 and QLQ-BR45 sexual scales for cancer survivors and cancer-free controls are presented in five age groups (18–39, 40−49, 50−59, 60−69, and ≥70 years) in Table 2 and Figure 1. Cancer survivors in all age groups had lower mean scores on all functional scales except for the *Importance of sexual activity,* which was equal in the two samples, and *Communication with professionals*, which was low in both samples, but slightly higher in cancer survivors. The symptom severity was generally higher in cancer survivors than in cancer-free controls, where the impact of fatigue on sexual life (both sexes), and vaginal dryness and discomfort (women only) were the most prominent symptoms in cancer survivors (Table 2 and Figure 1).

**Table 2:** Mean values for EORTC QLQ-SHQ22 and for the sexual scales in QLQ-BR23 and QLQ-BR45 by age groups in cancer survivors and cancer-free controls

		Cancer survivors						Cancer-free controls					

		All	18–39 years	40–49 years	50–59 years	60–69 years	70–79 years	All	18–39years	40–49 years	50–59 years	60–69 years	70–79 years
**Functional scales QLQ-SHQ22**													
Sexual satisfaction	M	46.9	55.1	40.8	51.3	46.2	45.8	56.2	59.0	58.1	55.4	53.4	50.1
(n=440/n= 4609)	SD	25.8	24.0	21.0	30.9	25.2	24.9	25.7	24.6	25.5	26.7	25.6	26.4
Importance of Sexual Activity	M	46.8	66.6	53.3	57.1	47.7	37.6	54.9	59.6	60.5	55.2	48.8	39.6
(n=446/n=4637)	SD	32.5	32.6	33.4	36.4	30.4	30.0	33.1	32.3	31.7	32.4	32.7	34.0
Libido	M	54.2	66.7	52.2	53.0	56.5	50.9	70.2	75.0	72.7	69.2	66.8	58.3
(n=444/n=4623)	SD	35.8	36.8	39.8	38.8	34.2	35.2	31.7	29.9	31.4	32.0	31.7	33.8
Treatment	M	53.3	71.4	54.9	52.6	51.9	52.9	82.3	85.3	83.4	81.7	79.8	76.0
(n=190/n=1242)*	SD	41.1	40.5	44.0	42.3	39.9	42.2	29.7	27.5	29.1	29.4	31.3	34.3
Communication with professionals	M	10.6	9.7	10.8	10.3	11.0	10.5	5.9	8.2	4.0	5.0	5.2	4.7
(n=439/n=4614)	SD	20.2	18.3	18.0	21.2	20.0	20.9	15.3	18.1	12.2	14.7	13.3	14.0
Security with partner	M	70.1	62.5	63.8	77.2	70.3	68.9	78.4	75.9	82.5	80.4	78.5	73.6
(n=344/n=3566)	SD	32.4	36.3	30.0	30.9	34.8	30.0	27.1	28.8	24.2	26.2	27.0	28.0
Confidence erection	M	51.8	75.0	75.0	69.8	46.1	44.3	68.3	75.7	75.0	66.8	62.0	53.2
(n=168/n=1849)	SD	36.3	38.8	20.7	34.8	36.4	34.5	33.5	32.3	31.4	33.2	33.5	33.0
Masculinity	M	67.8	77.8	83.3	74.4	64.6	65.9	81.6	86.1	87.8	81.1	80.8	66.2
(n=115/n=622)*	SD	35.0	27.2	27.9	38.9	36.6	34.1	29.2	27.5	24.2	29.5	29.4	32.7
Femininity	M	64.6	62.5	46.2	69.7	69.7	63.5	83.7	84.9	80.5	81.1	87.5	89.2
(n=97/n=571)*	SD	37.2	45.2	34.8	34.0	35.7	40.7	28.6	27.8	32.8	28.9	23.4	26.1
**Symptom scales QLQ-SHQ22**													
Sexual pain	M	9.0	6.5	12.1	9.2	10.7	6.9	6.3	8.3	4.8	5.5	6.1	4.9
(n=429/n=4591)	SD	19.2	12.3	25.7	19.2	20.6	17.1	15.1	16.6	12.7	14.0	15.2	14.8
Worry Incontinence	M	22.3	8.6	23.7	19.7	21.9	25.4	11.2	7.5	10.8	13.2	12.6	17.7
(n=444/n=4618)	SD	30.8	25.1	32.4	31.5	30.1	31.4	21.5	18.2	21.6	22.8	22.0	25.0
Fatigue	M	32.1	40.0	43.3	35.6	28.1	31.5	26.1	28.5	28.4	27.7	21.7	18.3
(n=439/n=4607)	SD	33.9	34.0	34.1	37.4	31.8	34.0	30.4	31.9	30.8	30.8	27.8	25.7
Vaginal Dryness	M	32.6	23.1	41.0	33.3	36.2	27.0	21.7	18.2	15.9	24.8	31.2	34.4
(n=149/n=2016)	SD	30.6	28.5	43.4	31.4	30.8	24.6	26.5	24.1	23.2	28.7	28.7	29.5
**Functional scales QLQ-BR23/45**													
Body Image	M	77.7	68.6	67.9	71.1	78.1	83.3	77.4	70.5	76.5	78.9	84.0	87.0
(n=448/n=4631)	SD	23.7	22.8	22.1	27.5	23.4	21.3	24.9	27.6	24.3	23.8	20.8	18.1
Sexual Functioning	M	44.7	52.8	40.8	45.5	46.0	42.8	53.4	56.5	55.8	52.3	49.8	46.9
(n=446/n=4631)	SD	25.2	22.9	22.7	27.2	24.3	25.8	25.5	24.8	24.5	25.9	25.2	27.2
Sexual Enjoyment	M	67.1	66.7	60.0	71.3	65.5	68.6	73.8	73.1	76.7	74.3	72.5	71.1
(n=320/n=3868)	SD	27.2	29.8	27.2	30.9	27.2	24.7	26.2	26.9	25.1	25.9	25.6	26.9
**Symptom scales QLQ-BR45**													
Endocrine Therapy Symptom	M	24.4	18.6	28.7	27.2	22.9	24.8	18.4	14.5	17.5	21.2	21.0	22.6
(n=451/n=4650)	SD	19.0	19.2	25.0	21.0	17.7	18.0	17.1	15.1	17.3	18.5	17.1	17.0
Endocrine Sexual Symptoms	M	25.7	20.5	35.8	23.8	30.2	18.5	17.5	16.9	12.3	18.7	22.5	24.3
(n=149/n=2021)	SD	26.1	27.3	38.4	25.0	27.7	15.6	20.5	19.8	16.5	22.3	22.3	23.2

* Respondents without challenges related to treatment had the option to answer, “not relevant”.

Adjusted analyses comparing sexual health scores between cancer survivors and cancer-free controls (Table 3) confirm the descriptive pattern in Figure 1. Cancer survivors scored significantly lower on all sexual health outcomes describing function, and higher on all sexual health outcomes describing symptoms compared to the cancer-free controls, with only one exception – an active sex life was equally important to the participants.

**Table 3:** Sexual health outcomes by the EORTC SHQ22, QLQ-BR23 and QLQ-BR45 in cancer survivors compared to cancer-free controls

	Intercept	Cancer survivors		Age		Sex		Morbidity		Weight satisfaction	
		Coeff.	p-value	Coeff.	p-value.	Coeff.	p-value	Coeff.	p-value	Coeff.	p-value
**Functional scales QLQ-SHQ22**											
Sexual satisfaction	61.14	-4.81	<0.001	0.01	0.849	4.87	<0.001	-9.49	<0.001	-6.61	<0.001
Importance of Sexual Activity	49.18	0.73	0.652	0.85	<0.001	14.10	<0.001	-4.39	<0.001	-4.73	<0.001
Libido	75.97	-8.95	<0.001	-0.20	0.048	16.28	<0.001	-10.32	<0.001	-6.20	<0.001
Treatment	97.62	-26.06	<0.001	-0.43	0.034	0.77	0.642	-15.45	<0.001	-4.96	0.017
Communication with professionals	10.85	4.97	<0.001	-0.38	<0.001	-0.18	0.683	4.09	<0.001	0.24	0.670
Security with partner	74.35	-6.60	<0.001	0.74	<0.001	-5.46	<0.001	-9.19	<0.001	-5.87	<0.001
Confidence erection	76.17	-6.52	0.017	0.39	0.030	-	-	-11.34	<0.001	-6.32	0.002
Femininity	95.95	-21.59	<0.001	-0.61	0.032	-	-	-9.36	<0.001	-10.60	<0.001
Masculinity	95.70	-7.87	0.010	-0.50	0.850	-	-	-19.74	<0.001	-6.68	0.018
**Symptom scales QLQ-SHQ22**											
Sexual pain	12.86	2.80	<0.001	-0.31	<0.001	-7.39	<0.001	5.18	<0.001	0.65	0.234
Worry Incontinence	4.68	6.91	<0.001	0.25	0.001	-6.58	<0.001	5.86	<0.001	5.00	<0.001
Fatigue	20.99	7.97	<0.001	0.69	<0.001	-10.67	<0.001	16.96	<0.001	4.01	<0.001
Vaginal dryness	18.83	4.96	0.034	-0.21	0.113	-	-	3.37	0.010	-2.21	0.138
**Functional scales QLQ-BR23/45**											
Body Image	70.74	-3.81	<0.001	0.19	0.006	11.96	<0.001	-11.09	<0.001	-20.43	<0.001
Sexual Functioning	54.09	-3.87	0.002	0.06	0.431	11.71	<0.001	-5.74	<0.001	-3.71	<0.001
Sexual Enjoyment	72.86	-4.74	0.002	0.17	0.080	5.28	<0.001	-8.30	<0.001	-3.68	0.001
**Symptom scales QLQ-BR45**											
Endocrine Therapy Symptoms	6.33	1.48	0.050	0.44	<0.001	-4.81	<0.001	16.27	<0.001	8.73	<0.001
Endocrine Sexual Symptoms	19.51	5.30	0.004	-0.42	<0.001	-	-	4.03	<0.001	-1.37	0.239

Estimates were derived from multiple linear regression analyses adjusted for age, sex, morbidity, and satisfaction with weight. Coding: age in years above 18, sex (male=0, women =1), Morbidity (no morbidity=0, one or more morbidities affecting daily functioning/activities=1), Weight satisfaction (not overweight= 0, overweight=). For illustration, the following equation estimates the sexual satisfaction in a woman aged 50, with one or more health condition affecting daily functioning and self-evaluation of being too heavy (overweight). Sexual satisfaction (predicted) = 61.14 + sex* 4.87 + (age-18) *0.01 + (age-18) ^2 * –0.00 + morbidity * - 9.49 + Weight * - 6.61. Sexual satisfaction (predicted) = 61.14 + 0 (female)*4.87 + (50-18) * 0.01 + (50-18) ^2 *-0.00 + 1 (morbidity) *– 9.49 + 1 (Overweight) * -6.61 = 45.36.

The most prominent differences appeared in the functional scales *Femininity* (mean difference 21.6, *p* < 0.001), *Treatment* (mean difference 26.1, *p* < 0.001)*, Libido* (mean difference 9.0, *p* < 0.001), and the symptom scales *Fatigue* (mean difference 8.0, *p* < 0.001) and *Worry of Incontinence* (mean difference 7.0, *p* < 0.001) (Table 3).

### Differences in sexual health between short-term and long-term cancer survivors

Long-term cancer survivors scored significantly higher than short-term cancer survivors on two SHQ22 scales: *Sexual satisfaction* (mean difference 7.3, *p* = 0.018) and *Importance of sexual activity* (mean difference 8.8, *p* = 0.010), as well as the BR23/45 scale for *Sexual functioning* (mean difference 8.8, *p* = 0.002). However, for most of the sexual scales, no significant differences were observed between short-term and long-term cancer survivors (Table 4).

**Table 4:** The impact of time after diagnosis on the EORTC sexual health outcomes in cancer survivors.

	Intercept	Time after diagnosis		Age		Sex	

		Coeff.	p-value	Coeff.	p-value.	Coeff.	p-value
**Functional scales QLQ-SHQ22**							
Sexual satisfaction	44.06	7.30	0.018	-0.36	0.513	3.14	0.217
Importance of Sexual Activity	39.56	8.80	0.010	0.43	0.489	21.24	<0.001
Libido	52.65	5.52	0.179	-0.32	0.666	12.95	<0.001
Treatment	137.5	1.01	0.889	-4.14	0.043	-5.05	0.437
Communication with professionals	1.27	3.66	0.126	-0.06	0.873	4.73	0.016
Security with partner	34.42	3.02	0.494	1.84	0.030	-5.47	0.139
Confidence erection	79.71	-2.58	0.726	0.11	0.930	-	-
Femininity	35.36	14.11	0.127	-0.30	0.833	-	-
Masculinity	69.08	3.17	0.697	-0.09	0.950	-	-
**Symptom scales QLQ-SHQ22**							
Sexual pain	5.57	2.19	0.325	0.325	0.413	-13.78	<0.001
Worry Incontinence	0.85	1.98	0.585	0.60	0.362	-3.23	0.285
Fatigue	61.00	-0.53	0.893	-9.72	0.176	-10.35	0.002
Vaginal dryness	2.49	-6.75	0.311	1.21	0.907	-	-
**Functional scales QLQ-BR23/45**							
Body Image	66.38	-0.50	0.849	-0.30	0.522	8.95	<0.001
Sexual Functioning	34.52	8.81	0.002	-2.50	0.628	12.68	<0.001
Sexual Enjoyment	69.85	1.45	0.722	-0.36	0.570	3.61	0.254
**Symptom scales QLQ-BR45**							
Endocrine Therapy Symptoms	17.28	-3.03	0.164	0.77	0.055	-5.13	0.005
Endocrine Sexual Symptoms	-3.42	9.56	0.092	1.01	0.211	-	-

Estimates were derived from multiple linear regression analyses adjusted for age and time after cancer diagnosis. Coding: age in years above 18, sex (male=0, women =1), Time after diagnosis (short-term survivor 0-2 years = 0, long-term survivor 2+ years = 1), and age squared (age^2).

### Sexual health in survivors of female- and male-specific cancers compared to cancer-free controls

Female-cancer survivors generally had poorer sexual health than women without cancer. The largest mean difference was found in the functional scales *Treatment* (−27.3, *p* < 0.001), *Femininity* (−19.8, *p* < 0.001) and *Libido* (−6.9, *p* = 0.026), and in the symptoms *Fatigue* (8.1, *p* = 0.003), *Sexual pain* (*7.4, p < 0.001) and Vaginal dryness* (6.9, *p* = 0.024). (Table 5).

**Table 5:** Sexual health outcomes by the EORTC SHQ22, QLQ-BR23 and QLQ-BR45 in female cancer survivors* compared to female cancer-free controls.

	Intercept	Female cancer survivors*		Age		Morbidity		Weight satisfaction	

		Coeff.	p-value.	Coeff.	p-value	Coeff.	p-value	Coeff.	p-value
**Functional scales QLQ-SHQ22**									
Sexual satisfaction	61.42	-4.26	0.081	0.07	0.543	-8.32	<0.001	-5.17	<0.001
Importance of Sexual Activity	54.93	-0.32	0.914	0.62	<0.001	-4.09	0.003	-3.81	0.014
Libido	79.70	-6.90	0.026	-0.60	<0.001	-8.10	<0.001	-6.17	<0.001
Treatment	97.69	-27.28	<0.001	-0.99	<0.001	-11.33	<0.001	-3.85	0.192
Communication with professionals	12.73	2.53	0.106	-0.43	<0.001	3.40	<0.001	0.81	0.310
Security with partner	72.22	-4.68	0.110	0.78	<0.001	-8.44	<0.001	-6.31	<0.001
Femininity	94.93	-19.78	<0.001	-0.56	0.055	-8.69	<0.001	-11.03	<0.001
**Symptom scales QLQ-SHQ22**									
Sexual pain	14.30	7.39	<0.001	-0.49	<0.001	6.27	<0.001	1.62	0.079
Worry Incontinence	3.08	1.91	0.412	0.41	<0.001	4.67	<0.001	6.66	<0.001
Fatigue	20.39	8.10	0.003	0.67	<0.001	17.93	<0.001	4.42	<0.001
Vaginal dryness	18.89	6.88	0.024	-0.22	0.103	3.40	0.009	-2.18	0.145
**Functional scales QLQ-BR23/45**									
Body Image	69.01	-5.00	0.027	0.28	0.011	-10.79	<0.001	-25.31	<0.001
Sexual Functioning	57.98	-4.80	0.042	0.20	0.075	-5.31	<0.001	-2.54	<0.001
Sexual Enjoyment	71.69	-3.04	0.326	0.23	0.094	-7.11	<0.001	-1.64	0.276
**Symptom scales QLQ-BR45**									
Endocrine Therapy Symptoms	4.60	1.00	0.481	0.54	<0.001	16.25	<0.00	10.30	<0.001
Endocrine Sexual Symptoms	19.57	6.71	0.005	-0.43	<0.001	4.08	<0.001	-1.35	0.249

Estimates were derived from multiple linear regression analyses adjusted for age, morbidity, and weight satisfaction. Coding: age in years above 18, Morbidity (no morbidity=0, one or more morbidities affecting daily functioning/activities=1), Weight (not overweight= 0, overweight=), Female cancer types (no cancer= 0, cancer =1).

* Female cancer survivors; breast-, ovarian-, cervix-, and uterus cancer.

Male cancer survivors also had worse sexual health than male cancer-free controls. The largest mean differences compared to cancer-free controls were found in the functional scales *Treatment* (−34.8, *p* < 0.001), *Libido* (−18.8, *p* < 0.001*), Masculinity* (−16.9, *p* < 0.001)*, Confidence with Erection* (−15.6, *p* < 0.001), *Sexual satisfaction* (11.07, *p* < 0.001), *insecurity with partner* (−9.7, *p* = 0.004) and in the symptom scale *Worry of Incontinence* (18.7, *p* < 0.00) (Table 6).

**Table 6:** Sexual health outcomes by the EORTC SHQ22, QLQ-BR23 and QLQ-BR45 in male cancer survivors* compared to male cancer-free controls.

	Intercept	Male cancer survivors*		Age		Morbidity		Weight satisfaction	

		Coeff.	p-value.	Coeff.	p-value	Coeff.	p-value	Coeff.	p-value
**Functional scales QLQ-SHQ22**									
Sexual satisfaction	65.50	-11.07	<0.001	0.10	0.393	-10.99	<0.001	-8.45	<0.001
Importance of Sexual Activity	53.81	-0.37	0.910	1.29	<0.001	-5.45	<0.001	-5.48	0.002
Libido	86.66	-18.82	<0.001	0.30	0.030	-13.40	<0.001	-5.78	<0.001
Treatment	97.31	-34.80	<0.001	-0.08	0.788	-18.34	<0.001	-6.10	0.037
Communication with professionals	7.61	14.54	<0.001	--0.25	0.001	4.63	<0.001	-0.38	0.650
Security with partner	73.45	-9.70	0.004	0.57	0.002	-9.87	<0.001	-5.20	0.007
Confidence erection	76.40	-15.57	<0.001	0.01	0.046	-11.30	<0.001	-6.47	0.002
Masculinity	95.46	-16.92	<0.001	0.03	0.902	-19.58	<0.001	-6.09	0.030
**Symptom scales QLQ-SHQ22**									
Sexual pain	3.48	0.46	0.606	-0.11	0.009	3.73	<0.001	-0.33	0.497
Worry Incontinence	0.57	18.67	<0.001	0.06	0.522	7.42	<0.001	2.59	0.012
Fatigue	10.51	5.16	0.058	0.39	0.003	19.11	<0.001	4.79	0.001
**Functional scales QLQ-BR23**									
Body Image	85.40	-3.62	0.048	0.07	0.431	-11.22	<0.001	-14.09	<0.001
Sexual Functioning	59.73	-7.62	0.002	0.45	<0.001	-6.63	<0.001	-4.9	<0.001
Sexual Enjoyment	79.43	-9.65	0.003	0.09	0.479	-9.91	<0.001	-6.18	<0.001
**Symptom scales QLQ-BR45**									
Endocrine Therapy Symptoms	4.25	-0.57	0.703	0.28	<0.001	16.53	<0.001	6.47	<0.001

Coding: age in years above 18, Morbidity (no morbidity=0, one or more morbidities affecting daily functioning/activities=1), Weight (not overweight= 0, overweight=1), Male cancer types (no cancer= 0, cancer =1).

* Male cancer survivors; prostate- and testicular cancer.

For both female and male cancer survivors, the *Importance of Sexual Activity* was of equal importance compared to their cancer-free controls.

## Discussion

Sexual health was significantly poorer in Norwegian cancer survivors than in age-matched cancer-free controls, which is consistent with a previous study of breast cancer patients [26]. However, in cancer populations with other diagnosis, comparisons to reference norms are lacking [27]. The lack of sufficient communication about sexual health topics with healthcare professionals was common for all individuals. Despite sexual challenges, having an active sex life was equally important to cancer survivors as to healthy controls.

The deteriorated sexual health in cancer survivors aligns with the high prevalence of sexual dysfunction shown in previous studies [1–4]. Treatment had a larger impact on sexual activity in cancer survivors compared to controls with other kinds of diseases and/or treatments. This finding is expected as symptoms from cancer treatment, such as fatigue, nausea, and insomnia, are known to have significant impact on sexual health [1, 28]. Cancer treatment itself may directly or indirectly impair sexual health. For example, women with gynecological cancers can experience vaginal thinning, clitoral atrophy or decreased vaginal elasticity [29], while breast cancer survivors may experience sexual pain and premature menopause [30]. Prostate cancer patients may experience decreased potency due to damage of the vasculature of the cavernous nerves and the penile bulb [13]. Testicular cancer patients may experience neuropathy, nerve disconnection, and interruption of normal blood supply to maintain erection, particularly if several treatment modalities are combined [4]. Such side effects may explain why cancer survivors in our study felt less feminine or masculine and reported lower libido compared to cancer-free controls. Impaired sexual health in short-term survivors was not significantly improved in long-term survivors, which is consistent with findings from a previous longitudinal study of survivors and population controls [26]. Sexual health challenges are shown to be more pronounced for female cancer survivors under 50 years [31, 32] and seem to persist for a long term in both breast and gynecological cancer survivors [26, 33]. Notably, if sexual problems persist for a long time, they may cause significant impairment in survivors’ quality of life [17]. Identifying and communicating about sexual health challenges are therefore especially important in the prevention of sexual dysfunction [33, 34].

To support meaningful interpretation of patient reported outcomes (PROs), it is essential to compare PROs from cancer survivors with normative data [18, 35]. This is particularly important for sexual health challenges, as these may be attributed to natural aging, effects of cancer or its treatment [27]. Our study found that cancer survivors reported poorer sexual health outcomes than controls, with high and moderate clinical significance [24] across several domains. These domains include the impact of treatment on their sexual life, libido, insecurity with partner, masculinity, femininity, and vaginal dryness. Sexual health impairments were more pronounced in cancer survivors younger than 50 years and were often specific to either females or males. This underscores the importance of interpreting PROs by age and sex.

Female cancer survivors experienced endocrine symptoms such as vaginal dryness, reduced libido and sexual pain, and their sexual activity was more influenced by fatigue and treatment than female cancer-free controls. Sexual problems related to sexual functioning, diminished lubrication, decreased libido, and problems with reaching orgasm and dyspareunia are congruent to previous reports among female cancer survivors [1, 29, 36]. Our findings are also in line with previous studies comparing sexual health in cancer survivors with cancer-free controls [26, 33].

Male cancer survivors reported significantly poorer sexual health than cancer-free controls in nearly all domains: lower libido, decreased masculinity, reduced confidence in erection and ability to satisfy their partner, and overall lower sexual satisfaction. The main concern among male cancer survivors was worries about incontinence. These findings align with known side effects such as erectile dysfunction [8, 14], loss of libido [8, 37], worry about incontinence [3, 4, 13] and psychological concerns related to manhood and losing function in a vital sexual organ [37, 38]. Interestingly and seldom reported, male cancer survivors appeared to have poorer sexual health compared to females with more dimensions affected and larger differences from their cancer-free counterparts. This finding is consistent with a previous study showing more fatigue, dyspnea, anxiety, and depression in male cancer survivors compared to their age-matched reference group [28].

Our study revealed low levels of communication with health care professionals regarding sexual topics, among both cancer survivors and cancer-free controls, as supported by studies on cancer survivors [39]. Notably, conversations with health care professionals were slightly higher among male cancer survivors, naturally seen in context with their high symptom burden. Improved communication about sexual health can reduce the likelihood of complex sexual morbidity in long-term survivors [33]. To improve symptom management and patient-clinician communication, collecting electronic patient-reported outcomes on sexual health could provide a solid foundation for meaningful conversations [40], and opportunity to compare scores with cancer-free controls [18].

Our study found that, despite facing significant sexual health challenges, being sexually active was equally important to both cancer survivors and cancer-free controls, as described in a previous study [33]. This highlights the importance of addressing sexual health as part of health-related quality of life and thus being a natural part of cancer survivorship care.

### Strengths and limitations

The EORTC QLQ-SHQ22 is the first cancer-specific stand-alone sexual health questionnaire that provides valid and reliable information on this under-communicated and overlooked health issue in a large and growing population. Our study is the first to use this newly validated instrument in a large cancer population of both men and women with different diagnoses. With an additional comparison to cancer-free controls, our study contributes to a comprehensive and nuanced documentation of sexual health in cancer survivors.

Since this study was based on data from the general population [18], we do not have supplementary clinical and treatment information on the cancer survivors within this population which limits our possibility to evaluate the generalizability of our cancer survivor sample. However, comparison to Norwegian cancer survivor population data from the Cancer Registry (suppl. Table I) showed similar age and sex distribution. Additionally, the self-reported nature of cancer incidence and diagnosis timing could result in inaccuracies. However, given the low risk of recall bias for previous cancer diagnoses, we consider this limitation to pose a minimal threat to the study’s reliability. Another limitation is the use of satisfaction with weight as a proxy for BMI. However, we believe that satisfaction with weight is highly correlated with BMI. In this context, we argue that subjective satisfaction with weight could influence sexual health more than the objective BMI and thus could be treated as a reliable confounder in specific analyses.

While a 5-year benchmark is commonly used to address long-term survivorship challenges, we opted for a 2-year cut-off to differentiate between short- and long-term symptoms. The sexual health challenges that persist beyond the 2-year benchmark, and is deviating from cancer-free controls, are arguably those who warrant attention.

## Conclusion and implications

The present study is the first to address sexual health in both female and male cancer survivors compared with age-matched cancer-free controls by using the EORTC questionnaire QLQ-SHQ22. Despite facing profound sexual challenges, having an active sex life was equally important to cancer survivors as to healthy controls. The low level of communication about sexual issues with health care providers reveals a significant potential for improvement in the follow-up procedures of cancer survivors. Therefore, educating health care providers on how to employ a combination of medical, psychological, and communicative strategies is crucial as it empowers patients to make informed decisions and can significantly impact their overall treatment experience and psychological well-being. This practice involves proactive communication, where healthcare workers initiate conversations about sexual health as a standard part of post-treatment care. Collecting electronic patient-reported health outcomes, including sexual health, and comparing them to reference values at clinical controls could be an efficient approach for addressing this issue and further scheduling patients into more personalized follow-up-programs depending on their needs. Establishing clinically significant difference values for the specific QLQ-SHQ22 can offer a more nuanced interpretation of sexual health among cancer survivors.

## Supplementary Material

Sexual health in female and male cancer survivors – compared with age-matched cancer-free controls in Norway

## Data Availability

The data are available on request to the corresponding author, randi.j.reidunsdatter@ntnu.no.
