# Supplementary material for: Sexual health in female and male cancer survivors – compared with age-matched cancer-free controls in Norway
Source: Acta Oncol. 2025 Mar 7;64:42451. doi: 10.2340/1651-226X.2025.42451 (PMC11905150; doi:10.2340/1651-226X.2025.42451)

Supplementary material has been published as submitted. It has not been copyedited, or typeset by Acta Oncologica

Suppl. Fig 1 presents Directed Acyclic Graphs (DAGs) used to identify pertinent confounders in the multivariable regression analysis (1a-c). Factors displayed with arrow pointing towards both variables/predictors and outcomes are considered confounders. Confounders are factors that affect both the variables/predictors and the outcome. Factors displayed with arrow pointing from variables/predictors to outcomes are considered mediators. Mediators are factors which may be influenced by variables/predictors and may affect outcomes and were therefore not adjusted for in the statistical analysis.

Suppl. Figure 1a The association between cancer and sexual health. Confounders included are age, weight, and morbidity.

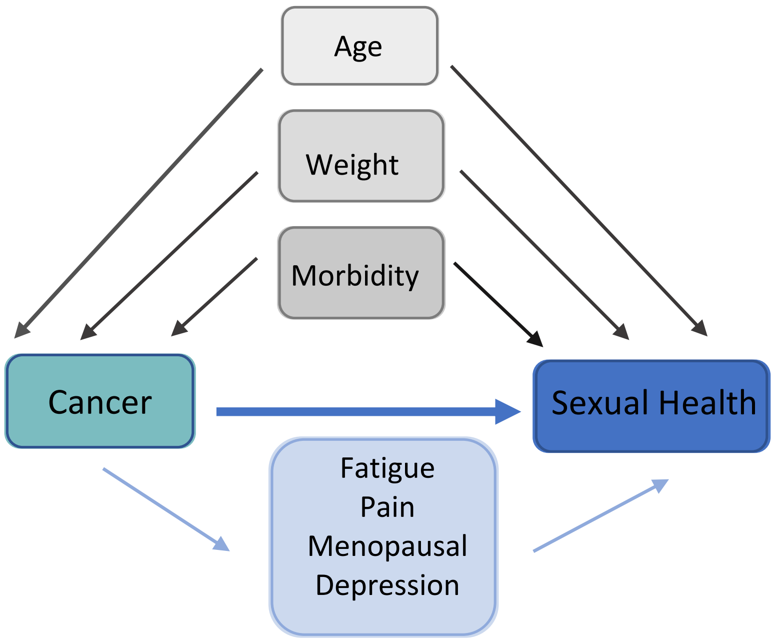

Suppl. Figure 1b The association between short-term versus long-term cancer survivors and sexual health. Confounder included is age.

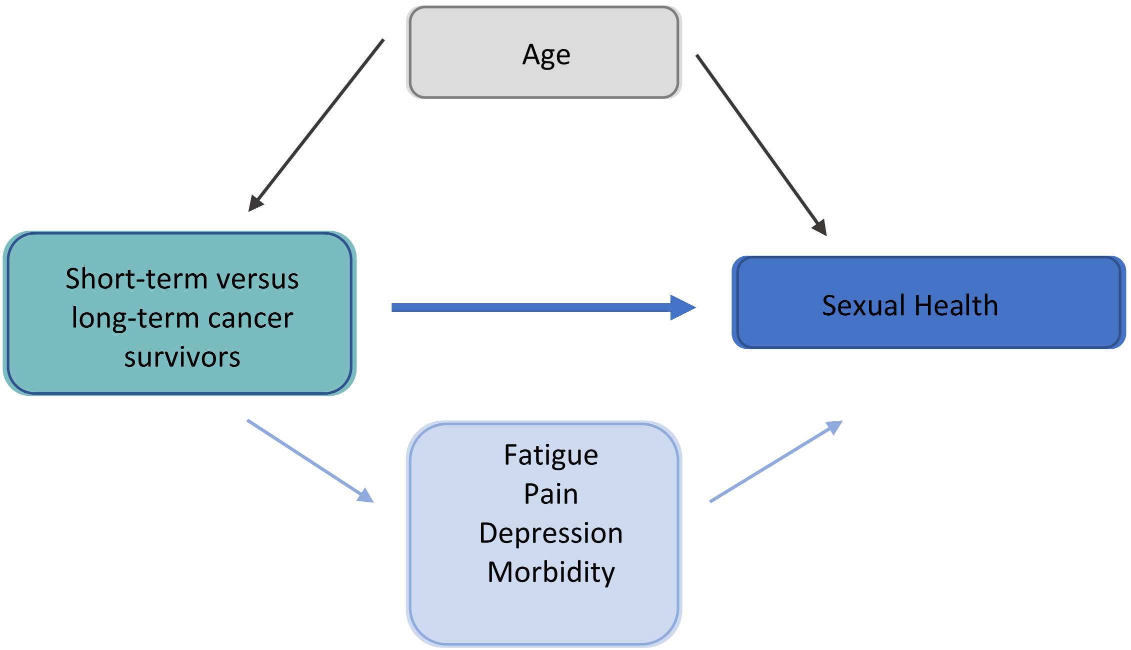

Suppl. Figure 1c The association between male/female specific cancer types and sexual health. Confounders included are age, weight, and morbidity.

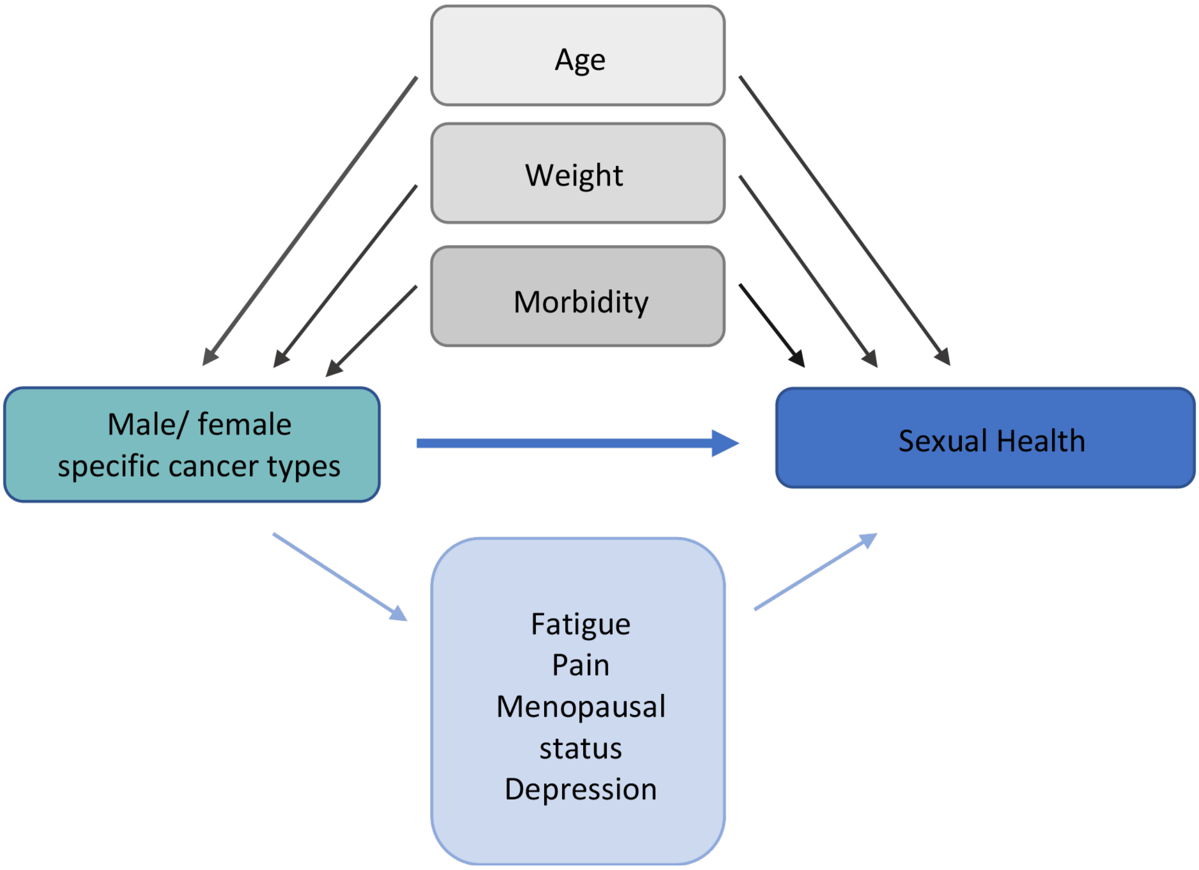

Supplement: Sexual health in female and male cancer survivors – compared with age-matched cancer-free controls in Norway [file AO-64-42451-s1.pdf]
